# A Fast and Efficient Approach to Obtaining High-Purity Glioma Stem Cell Culture

**DOI:** 10.3389/fgene.2021.639858

**Published:** 2021-07-06

**Authors:** Xin-Xin Han, Chunhui Cai, Li-Ming Yu, Min Wang, Dai-Yu Hu, Jie Ren, Meng-Han Zhang, Lu-Ying Zhu, Wei-Hua Zhang, Wei Huang, Hua He, Zhengliang Gao

**Affiliations:** ^1^Shanghai Key Laboratory of Craniomaxillofacial Development and Diseases, Shanghai Stomatological Hospital, Fudan University, Shanghai, China; ^2^Tongji University Cancer Center, Shanghai Tenth People’s Hospital, School of Medicine, Tongji University, Shanghai, China; ^3^School of Medicine, Jiaxing University, Jiaxing, China; ^4^Department of Neurosurgery, Changzheng Hospital, Second Military Medical University, Shanghai, China; ^5^Department of Neurosurgery, Third Affiliated Hospital of Second Military Medical University, Shanghai, China

**Keywords:** glioblastoma, cancer stem cell, neural stem cell, p53, tubulin beta 6 class V, SRY-box containing gene 2

## Abstract

Glioma is the most common and malignant primary brain tumor. Patients with malignant glioma usually have a poor prognosis due to drug resistance and disease relapse. Cancer stem cells contribute to glioma initiation, progression, resistance, and relapse. Hence, quick identification and efficient understanding of glioma stem cells (GSCs) are of profound importance for therapeutic strategies and outcomes. Ideally, therapeutic approaches will only kill cancer stem cells without harming normal neural stem cells (NSCs) that can inhibit GSCs and are often beneficial. It is key to identify the differences between cancer stem cells and normal NSCs. However, reports detailing an efficient and uniform protocol are scarce, as are comparisons between normal neural and cancer stem cells. Here, we compared different protocols and developed a fast and efficient approach to obtaining high-purity glioma stem cell by tracking observation and optimizing culture conditions. We examined the proliferative and differentiative properties confirming the identities of the GSCs with relevant markers such as Ki67, SRY-box containing gene 2, an intermediate filament protein member nestin, glial fibrillary acidic protein, and s100 calcium-binding protein (s100-beta). Finally, we identified distinct expression differences between GSCs and normal NSCs including cyclin-dependent kinase 4 and tumor protein p53. This study comprehensively describes the features of GSCs, their properties, and regulatory genes with expression differences between them and normal stem cells. Effective approaches to quickly obtaining high-quality GSCs from patients should have the potential to not only help understand the diseases and the resistances but also enable target drug screening and personalized medicine for brain tumor treatment.

## Introduction

Malignant glioma is a highly lethal brain cancer, and glioblastoma (GBM) is the most aggressive glioma type. The 5 year overall survival for GBM patients is less than 10% with the median survival of 14–16 months (Wankhede et al., 2012; Campos et al., 2016). It is difficult to characterize GBM subtypes posing a challenge to choose an appropriate therapeutic approach for patients due to a limited understanding of the underlying molecular biology (Campos et al., 2016; Karim et al., 2016). Therefore, there is an urgent need to understand the progression of GBM and to develop effective therapies for patients with GBM.

Glioblastoma is a highly heterogeneous tumor with various cellular subtypes (Aum et al., 2014; Soeda et al., 2015) and genetic properties (Sullivan et al., 2014; Li et al., 2015). The tumor mass is complex and contains mature cells, GSCs, and neural stem cells (NSCs). The concept of GSCs emerged in the early 2000s but remained to be fully characterized (Hemmati et al., 2003; Singh et al., 2003; Galli et al., 2004). As a subpopulation of tumor cells, GSCs are the driving force of tumor recurrence and can self-renew and differentiate (Taga and Tabu, 2020). Unlike GSCs, NSCs are tumor-tropic cells that can be used as vehicles to selectively deliver various anticancer agents to tumor sites (Mooney et al., 2018).

Recently, personalized therapies have been gaining momentum and are thought to significantly change the outcome for patients with GBM (Sundar et al., 2014; Graham-Gurysh et al., 2020; Samec et al., 2020; White et al., 2020). Ideally, therapy for patients with GBM would only kill GSCs but not affect NSCs. Therefore, it is important to separate GSCs from tissue samples after surgery and to identify the differences between GSCs and normal NSCs. The development of personalized therapy needs to occur quickly with high efficiency because of the high recurrence rate and short recurrence time. The key is to establish efficient protocols to quickly isolate pure GSCs from patient samples and do comparisons between GSCs and NSCs. This study was designed to establish a fast and efficient approach for obtaining high-purity glioma stem cells and to explore the morphological and genetic differences between GSCs and NSCs. We describe the features of GSC origin and compare differences in the properties and regulatory genes in GSCs and NSCs.

## Materials and Methods

### Surgical Glioma Samples

Surgical samples and basal data from human patients with glioma were obtained strictly according to Ethics Committee permission. Patients with glioma provided informed consent and donated their surgical specimens to the study. All the tumor samples were obtained and taken to the laboratory in time for follow-up treatment. Glioma tumors were categorized as grades II–IV using the WHO guidelines (Wesseling and Capper, 2018). After anonymous processing, surgical samples were coded by the research number.

### Plates Coating With Poly-L-Ornithine and Laminin

Culture plates were pre-covered with poly-L-ornithine (Sigma P3655) and laminin (Thermo Fisher 23017015) for hGSC culture and passage as previously described (Han et al., 2017). In brief, each 100 mm dish was treated with 10 ml 0.5 μg/ml poly-L-ornithine (10 mg/ml stock concentration dissolved in water) and maintained at room temperature for 16 h on a flat, clean tabletop. The following day, 1 × PBS (Hyclone SH30258.01) was used applied to wash the dishes after the poly-L-ornithine supernatant was discarded. Then, 10 ml 1 × PBS containing 5 μg/ml laminin was added to the dish and was left to keep infiltrating for at least 16 h at room temperature. Finally, the coated dishes were stored at −20°C. Before cell culture, the coated dishes were incubated at 37°C, and the supernatant was discarded.

### Glioma Stem Cells Derivation and Culture

After strictly following the ethical process, about 300 mg of tumor surgical samples was quickly transferred to a sterile 50 ml centrifuge tube and brought to the biosafety cabinet of the laboratory. The surgical samples were carefully removed with sterile forceps and were placed into the sterile 100 mm Corning cell culture dish. Surgical specimens were washed with 1 × HBSS (Gibco 14170-112) at least six times. 1 × HBSS was pre-cold at 4°C. After each washing, the samples were fished into a new sterile culture dish, and new Hanks’ balanced salt solution (HBSS) was added for a full cleaning. Then, the samples were evenly divided into new 100-mm dishes. Open the lid, carefully remove the tumor tissue with sterile forceps, and place it on the lid of the sterile 100 mm Corning cell culture dish. Cut into small pieces with a sterile blade, and then, cut into mud shape with ophthalmic forceps. Muddy tissue samples were placed into centrifuge tubes containing 3 ml 1 U/ml Dispase II (Roche 04942078001) in DMEM/F12 (Gibco 11330-033) or DMEM (Gibco 11995-065). 1 ml blue tips were used to head to blow and mixed the digestive enzyme and muddy tissue thoroughly and slowly, and then carefully transfer them into 15 ml sterile centrifuge tube. The tubes were incubated at 37°C in a water-bath for 30 min to allow digestion. During the period, the centrifuge tube can be taken out to check the digestion condition, and it is usually shaken evenly every 10 min. Be careful not to over digest. After digestion, tissues were centrifuged at 1,000 g for 3 min, and the supernatant was discarded carefully with a 1 ml blue tip. Cells suspended in 3 ml DMEMF12 or DMEM. Re-suspended cells were centrifuged again at 1,000 g for 3 min, and the supernatant was also discarded carefully with a 1 ml blue tip. The precipitate was blown no more than eight times each time with only 1 ml tips. Finally, the precipitate was suspended in DMEM/F12 (Gibco 11330-033)/N2 (Gibco 17502-048)/B27 (Gibco 17504-044)/GlutaMAX (Gibco 35050061)/bFGF (HumanZyme HZ-1272)/epidermal growth factor (EGF; R & D Systems 236-EG)/heparin (Sigma H3393)/penicillin/streptomycin (Gibco C14-15070-063) medium or DMEM/10% FBS (Gibco 10099)/GlutaMAX/penicillin/streptomycin medium. Fibroblast growth factor (FGF), EGF, and heparin concertations were 20 ng/ml. Cells were seeded onto coating with poly-L-ornithine and laminin or non-coated plates.

### Immunofluorescence Staining

Human glioma stem cells (hGSCs) were assessed using staining assays as described previously (Han et al., 2017). In brief, cells were grown for 3 or 7 days in optimized culture conditions and fixed with 4% paraformaldehyde for 12 min at room temperature. Then, cells were permeabilized with 2.5% Triton X-100 (Sigma V900502) in PBS and incubated for 15 min at room temperature. Then, we discarded the supernatant and blocked non-specific reactions with 5% bovine serum albumin (Solarbio, A8010) in 1 × PBS for 1.5 h at room temperature. Cells were incubated with primary antibodies to Sox2 (Goat, Santa Cruz sc-17320), nestin (Mouse, Millipore MAB5326), glial fibrillary acidic protein (GFAP; Rabbit, Abcam ab7260), S100-beta (Mouse, Sigma S2352), or Ki67 (Rabbit, Thermo Fisher 14-5698-82) for 2 days at 4°C. After three washes with 0.1% Tween-20 (Sigma 655206) in 1 × PBS, cells were incubated with secondary antibodies (Alexa Fluor 647-conjugated donkey anti-goat IgG 705-605-003, Alexa Fluor 488-conjugated donkey anti-mouse IgG 715-545-150, Alexa Fluor Cy3-conjugated donkey anti-rabbit IgG 711-165-152, Alexa Fluor Cy3-conjugated donkey anti-goat IgG 705-165-003, and Alexa Fluor 647-conjugated donkey anti-rabbit antibody 712-605-153, Jackson ImmunoResearch). Antibodies were dissolved in PBS containing 2.5% bovine serum albumin and incubated with cells at room temperature for 2 h. Cells were washed with 0.1% Tween-20 in PBS three times, and the nucleus stained with 4′,6-diamidino-2-phenylindole (Sigma D9542). Immunofluorescence staining images were captured using an inverted fluorescence microscope (Nikon TE2000).

### RNA Extraction, Sequencing, and Gene Expression Analysis

Passage 10 hGSCs were grown in DMEM/F12 medium on non-coated plates for 7 days. Passage 4 human neural stem cells (hNSCs) were cultured in the same medium on coated plates for 7 days. The medium was sucked away, and cells were washed once with PBS. Then, we added 2 ml of TRIzol (Invitrogen 15596026) and extracted the total RNA from the cells. For each cell type, two biological replicate RNA samples were collected for RNA sequencing. RNA integrity was determined using an Agilent 2100 Bioanalyzer (Agilent; Palo Alto, CA, United States). RNA quantity was determined using a NanoDrop (Thermo Fisher Scientific, Wilmington, DE). Poly-A-containing mRNA molecules were purified using ploy-T oligo-attached magnetic beads, fragmented, and primed for cDNA synthesis using the Illumina TruSeqTM RNA sample preparation kit (Illumina Inc., San Diego, CA) according to the manufacturer’s protocol. cDNA was converted into double-stranded DNA using the kit reagents. dsDNA was purified using AMPure XP beads (Invitrogen, Carlsbad, CA) and was end-repaired and A-tailed following Illumina’s protocol. After adapter ligation, PCR was used to enrich DNA fragments with adapter molecules on both ends and to amplify the amount of DNA in the library. The resulting molecular libraries were pooled and sequenced on a HiSeq 2500 sequencer (Illumina Inc., San Diego, CA). Then, the FPKM values were analyzed as the gene expression base. Differentially expressed gene (DEG) analysis was performed using online software (Morpheus, https://software.broadinstitute.org/morpheus) to identify up- and downregulated genes. Kyoto Encyclopedia of Genes and Genomes (KEGG) and Gene ontology (GO) analyses were also performed using an online database (g: Profiler; Reimand et al., 2007). The datasets presented in this study can be found in online repositories at https://bigd.big.ac.cn/gsa-human/browse/HRA000521, accession no: prjca004144.

## Statistical Analysis

All data were obtained from three or more replicates. Quantitative data are presented as mean ± standard deviation. Statistical analysis was performed using GraphPad Prism 7.0 (GraphPad Software, United States). For multiple comparisons, Student’s *t*-test was applied to check significance values. Value of *p* lower than 0.05 were considered statistically significant. Value of *p* were calculated from at least three independent experiments. Statistical significance was denoted as: ^*^p < 0.05, ^*^^*^p < 0.01, and ^*^^*^^*^p < 0.001. All error bars represent the standard deviation of the mean.

## Results

### Establishing an Efficient Protocol for Human Glioma Stem Cells Isolation and Expansion

**Figure 1:**
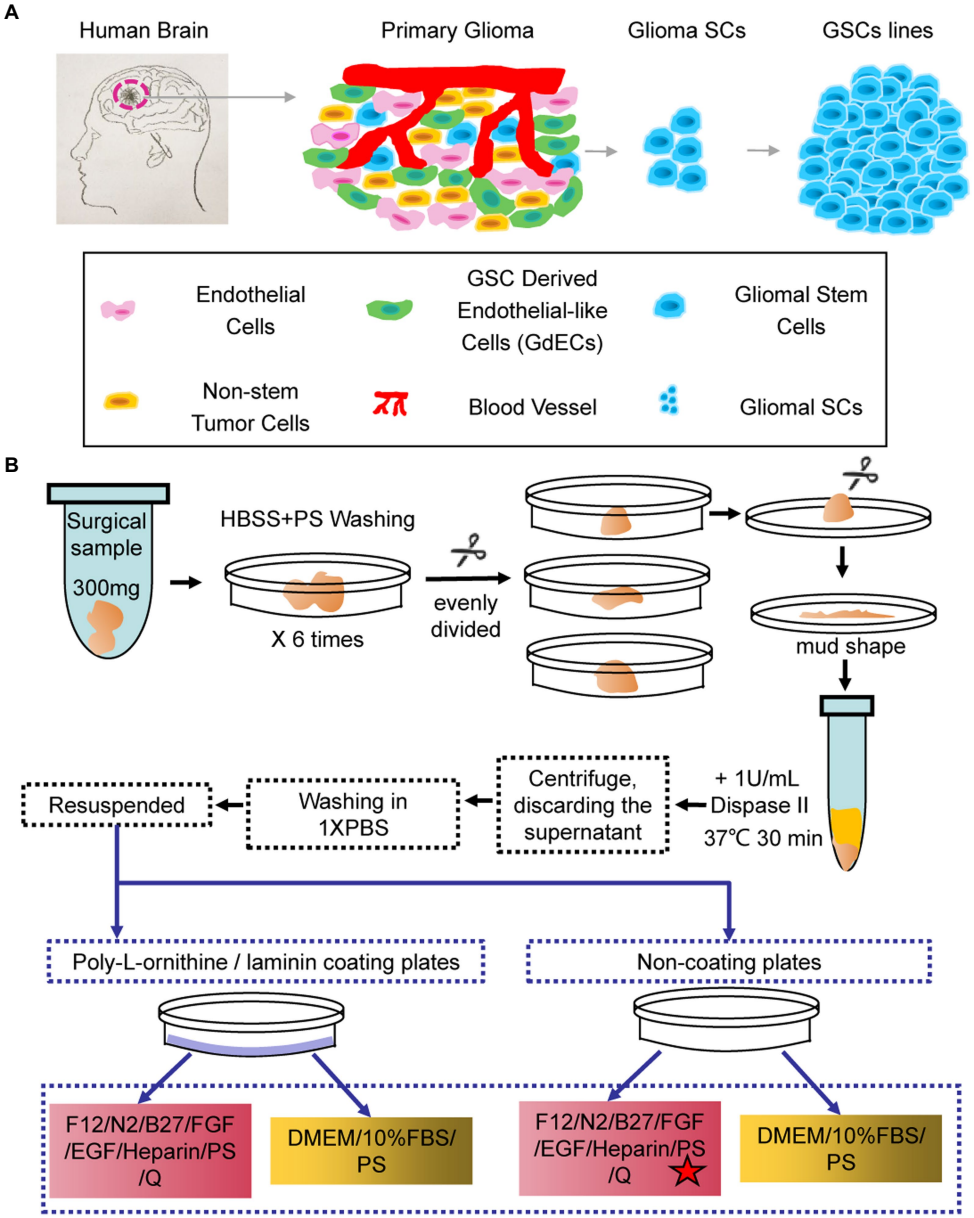
Schematic and flowchart describing how human glioma stem cell (hGSC) culture was derived. **(A)** hGSC isolation from glioma tissue and removal of other cells, including endothelial cells, glioma stem cells (GSCs)-derived endothelial-like cells, and non-stem tumor cells. **(B)** hGSC derivation procedures.

Primary GBM tissues contain many different types of cells including endothelial cells, non-stem tumor cells, GSC-derived endothelial-like cells, blood vessels, and hGSCs (Figure 1A). To better understand the molecular mechanisms of hGSCs, we aimed to develop a protocol to isolate the hGSCs from GBM tissues by primary culture. Various kinds of culture media and additives were used to culture GSCs in different studies (Zhang et al., 2020), and serum-free medium has been generally preferred (Lee et al., 2006). For comparison and for considering the heterogeneous nature of tumors, we decided to include both serum-supplemented medium and serum-free medium for our optimization. For the serum-free condition, we preferred DMEM/F12 medium over neural basal medium with N2 and B27 supplements. For serum-free medium, FGF, EGF, and heparin were added (Figure 1B). Primary cell cultures were also tested and maintained under both coated and non-coated conditions (Figure 1B). In total, we have shown optimization resulted from four different culture conditions (Figure 1B).

To derive GSC culture, patient surgical samples were washed in (HBSS)-PS six times and then chopped into small chunks. The chunks were digested with 30-min incubation in 1 U/ml Dispase II at 37°C. The disassociated cells were then spun down, washed, and re-suspended for primary culture at indicated culture conditions (Figure 1B) with medium changed every 3 days (Figure 1B).

### The Choice of the Optimal Method to Separate Human Glioma Stem Cells

**Figure 2:**
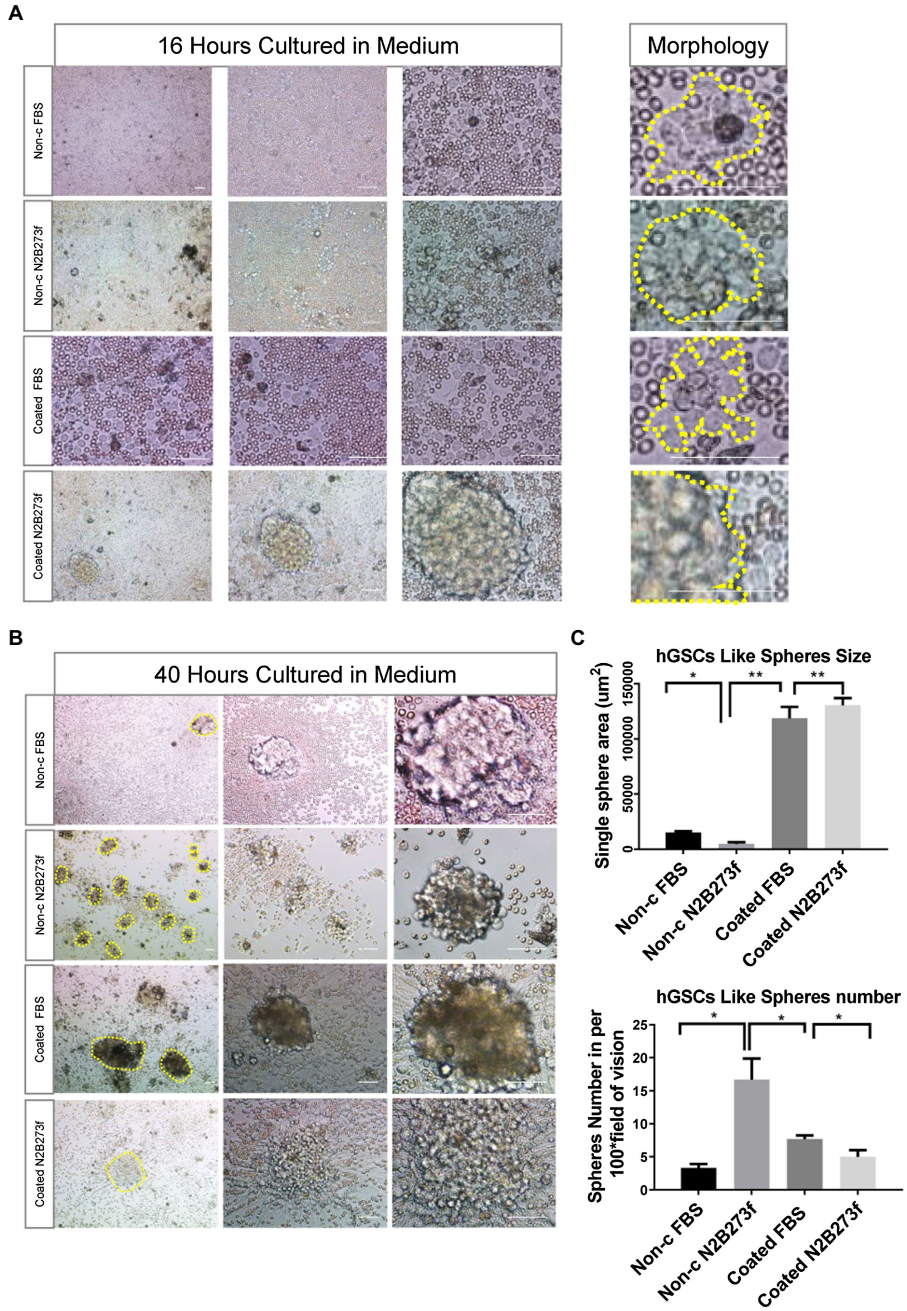
Separating hGSCs using different methods to identify an efficient and high-quality approach. **(A)** Representative photos of glioma tissue cell germination and morphology following culture in a different medium for 16 h. Yellow dotted line on the right shows the clonal morphology after 16 h germination under different culture conditions. Non-c FBS: culture cells on non-coated plates with DMEM + 10%FBS; Non-c N2B273f: culture cells on the non-coated plates with DMEM/F12 + N2B27 + 3 factors [Fibroblast growth factor (FGF), epidermal growth factor (EGF), and heparin]; coated FBS: culture cells on coated plates with DMEM + 10% FBS; coated N2B273f: culture cells on coated plates with DMEM/F12 + N2B27 + 3 factors (FGF, EGF, and heparin). **(B)** Primary glioma cells from glioma tissue cultured in a different medium for 40 h. **(C)** Single sphere area and spheres number of hGSCs in per 100x field of vision (non-c FBS with *n* = 3; Non-c N2B273f with *n* = 5; coated FBS with *n* = 3; and coated N2B273d with *n* = 3). Scale bar, 50 μm. Data are represented as mean ± SD. Student’s *t*-test; ^*^p < 0.05; ^*^^*^p < 0.01.

We monitored the culture closely through frequent observations after seeding. At 16 h, images of hGSCs were acquired at different magnifications (4×, 10×, and 20×) and small but visible cell clusters started to appear (Figure 2A). At 40 h, significant large cell spheres were shown in coated plates (Figure 2B). However, closer observations revealed that there were more small sphere colonies in the non-coated plates supplemented with 3F (Figure 2B). There are around 15–20 spheroids under each 4 × field in non-c N2B273f condition (Figure 2C). Further examination found that the morphology of cell spheres became irregular in the coated plates and in the non-coated plate with FBS. The larger spheres were likely resulted from the adherence and spreading of the spheres, an indication of fast proliferation and possible differentiation of spheres (Weiswald et al., 2015). In contrast, although they were significantly smaller, the small colonies in the non-coated plates supplemented with three factors were round with clear boundaries, reminiscent of healthy neurospheres of high proliferative potentials.

### Highly Efficient Derivations of Human Glioma Stem Cells From Glioblastoma Patients

**Figure 3:**
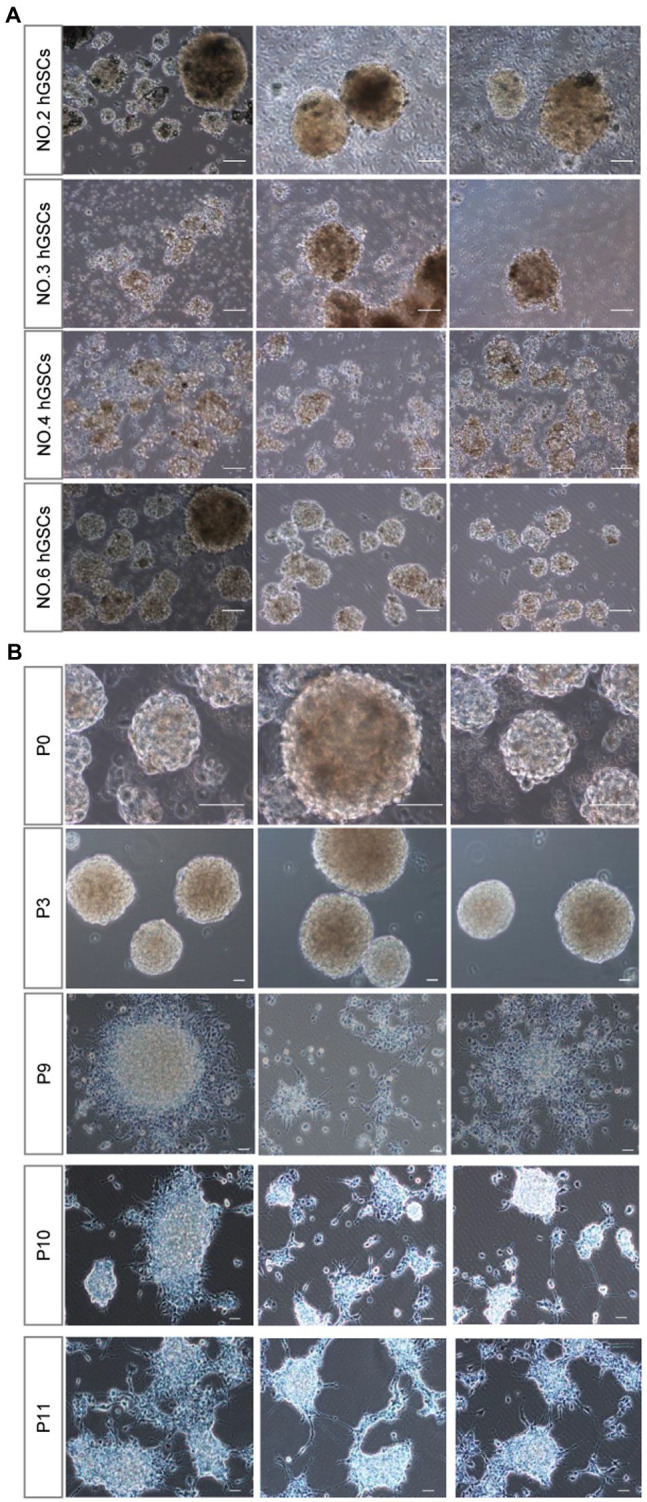
hGSCs isolated from different glioma tissues in P0 generation and different generations from P0 after passage. **(A)** Representative images of hGSCs isolated from different individual glioma tissues in P0 generation. hGSCs were cultured in optimized culture conditions for 7 days. **(B)** hGSCs can be passaged for over 10 generations. Scale bar, 50 μm.

Then, we sought to determine whether this culture system is suitable for surgical samples from different patients. Primary tumor cell cultures were successfully obtained for all the ten samples from non-selected patients. Among them, four hGSCs cell lines were successfully established (No. 2, No. 3, No. 4, and No. 6; Figure 3A) with the presence of abundant healthy spheres.

Considering the short median survival time for patients with GBM, personalized medicine requires a robust culture system that can be used to passage multiple generations of cells to produce sufficient numbers of hGSCs for drug selection tests. As shown in Figure 3B, we could routinely culture these cells continuously for at least over 9–10 passages. We did notice that the number of spheres decreased slightly and some of them became adherent around passage 10 cells. Nonetheless, we could continue the culture for all of them for many more passages. In conclusion, our culture system, using a non-coated plate with a medium supplemented with three factors, is a stable culture system that can maintain hGSCs for many generations and is suitable for various surgical samples.

### Molecular Confirmation and Characterization of Human Glioma Stem Cells

**Figure 4:**
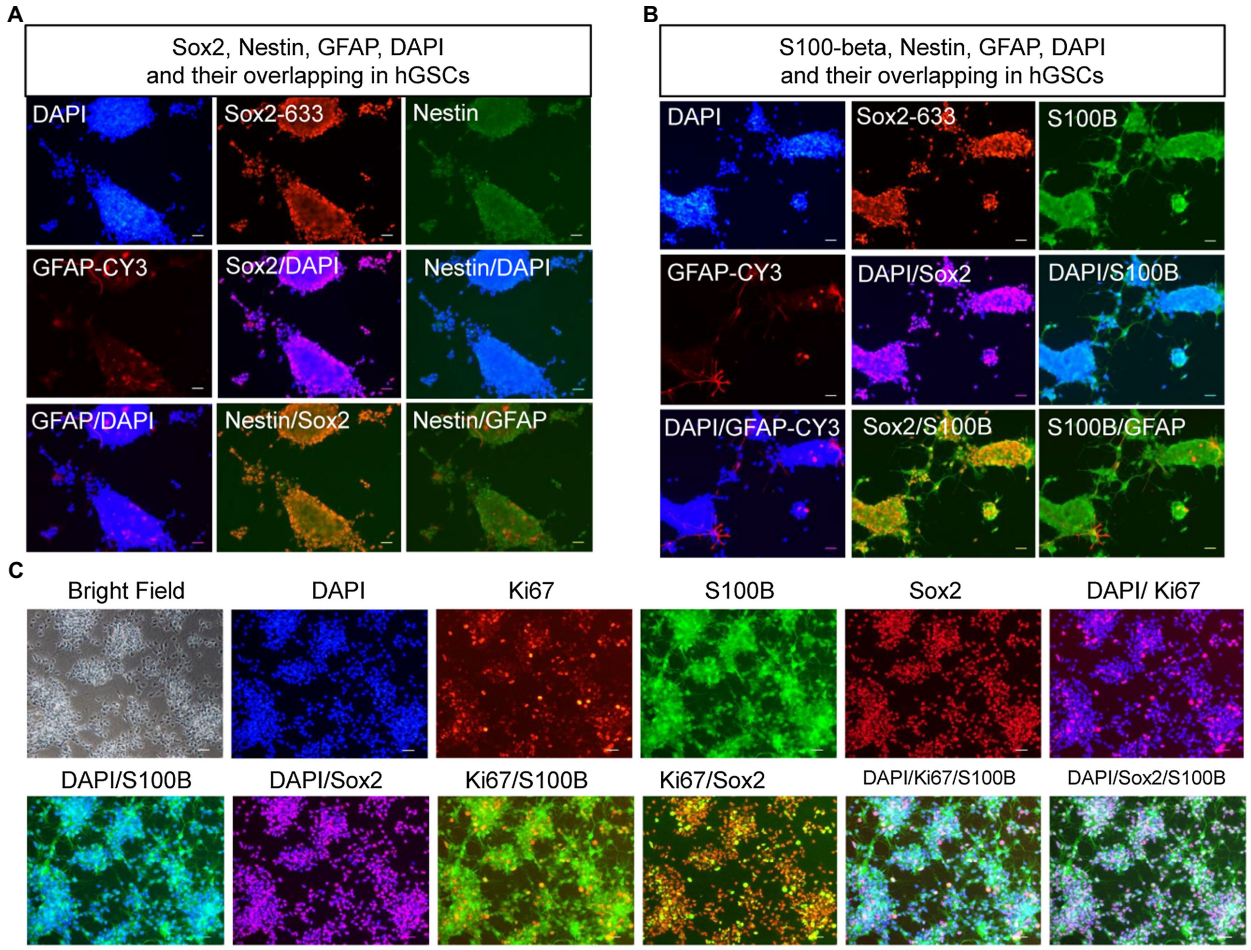
Confirmation and characterization of hGSCs. **(A–C)** hGSCs were fixed and immunostained with GFAP, Sox2, and nestin **(A)**; S100-beta, Sox2, and GFAP **(B)**; and Ki67, S100-beta, and Sox2 **(C)**. Scale bar, 50 μm.

To confirm the identities of the derived hGSCs, we stained them for SRY-box containing gene 2 (Sox2), nestin, glial fibrillary acidic protein (GFAP), and S100-beta (Figures 4A,B). As expected, all of them were highly expressed in the derived cells except GFAP which manifested a low expression in some of the cells. Furthermore, these cells also had strong proliferation potentials as demonstrated by their continuous passaging ability and Ki67 staining (Figure 4C). Together, these cellular analyses supported their identities as GSCs.

**Figure 5:**
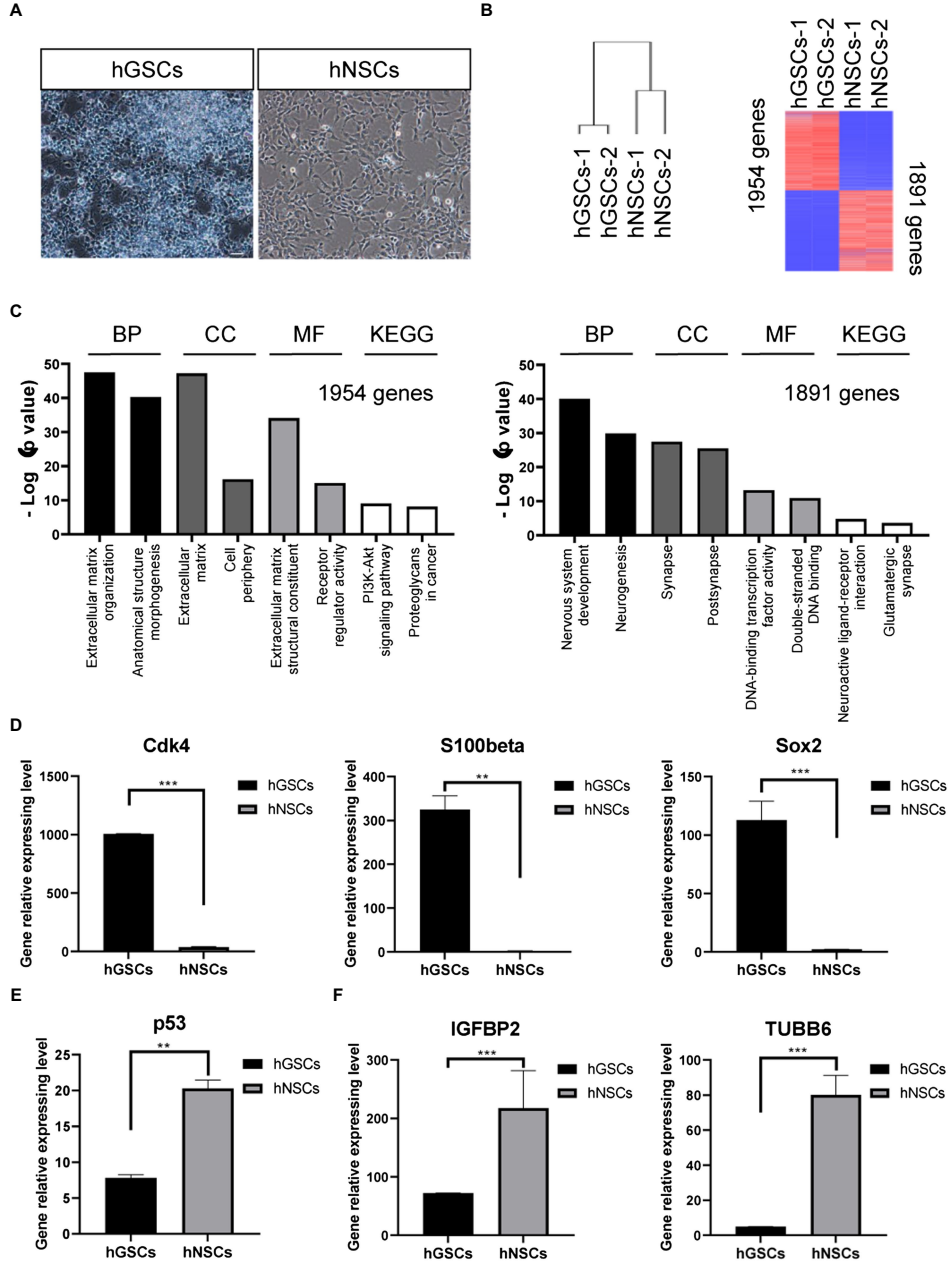
Molecular characterization and comparison of hGSCs and human neural stem cells (hNSCs). **(A)** hGSCs and hNSCs were morphologically different. **(B)** Gene clustering analysis of hGSCs and hNSCs and differential gene expression analysis of hGSCs and hNSCs. **(C)** The Gene ontology analysis (biological process, cell component and molecular functions) and KEGG analysis of upregulated genes (left) and downregulated genes (right). **(D)** The expression of Cdk4, S100-beta, and Sox2 was higher in hGSCs than that in hNSCs. **(E)** The expression of tp53 was lower in hGSCs than that in hNSCs. **(F)** Decreased IGFBP2 and TUBB6 expression was observed in hGSCs, compared to hNSCs. Data are represented as mean ± SD with *n* = 3 biological replicates.

To further confirm and characterize the identities of these GSCs, we carried out transcriptomic analysis by RNA sequencing. For comparison, we decided to include hNSCs. hGSCs and hNSCs were morphologically different. hGSCs formed spheres, while hNSCs cells exhibited strong adherent abilities (Figure 5A). Consistent with that, their molecular signatures were rather different too (Figure 5B). Different expression gene analysis showed 1954 genes had higher expression and 1891 genes had lower expression in hGSCs, compared to hNSCs (Figure 5B). GO and KEGG analyses revealed many pathways were differentially expressed in hGSCs or hNSCs including those related to the extracellular matrix and neurogenesis processes (Figure 5C). In this current study, for their relevance to GBM, we have chosen to focus on a number of genes related to cell cycle and stemness. As shown in Figure 5D, the expression level of cyclin-dependent kinase 4 (Cdk4), S100-beta, and Sox2 was all higher in hGSCs than that in hNSCs. In contrast, the expression of the p53 tumor suppressor gene was lower in hGSCs than that in hNSCs (Figure 5E). IGFB2 and tubulin beta 6 class V (TUBB6), two potential therapeutic targets in GBM treatment (Jiang et al., 2020), both displayed significantly lower expression in hGSCs than in hNSCs (Figure 5F).

## Discussion

Intensive efforts are under ways to predict potential therapeutic targets and to expand GBM treatment options. For example, DEG analysis has been used to identify differences in gene expression patterns between samples from patients with GBM and normal brain samples. Very recently, *CMPK2*, *CRLS1*, *PGS1*, *SLC22A5*, and *SOAT1* were identified as essential for GBM growth but non-toxic to remove from healthy brain tissue (Larsson et al., 2020). DEG, multivariate Cox, and microarray data K-M curve analyses also identified *SLC12A5*, *CCL2*, *IGFBP2*, and *PDPN* as independent predictors of survival in patients with GBM (Yang et al., 2020). Differentially expressed mitochondrial-focused gene (DEMFG) analysis also suggested microtubular TUBB6 as a potential therapeutic target in GBM (Jiang et al., 2020). Besides, Immunocore, an immune-infiltration-based signature, was also being considered as a potentially reliable prognostic and predictive tool for GBM (Tang et al., 2020). However, all these potential therapeutic strategies need to be further confirmed by experimentations before they could move forward to clinics. This is where a quick and robust drug screening system is required. A primary cell culture system like ours may fulfill both the speed and quality requirements. First, hGSC primary culture could be established timely within 1–3 days after surgery. Secondly, hGSCs can be maintained for more than 10 passages, and hence, our approach can help provide a large amount of cell material for drug screening and potential therapeutic target selection.

Brain organoids are a new approach for GBM modeling (Qian et al., 2016). Comparing to the two-dimensional culture of GSC cells, the organoid culture system acted as a 3D system may reproduce a better niche for GBM study (Gomez-Oliva et al., 2020). Organoid models are very useful for studies of essential tumor biology and also suitable for preclinical investigations, such as drug screening and analysis of antitumor effects accompanied by a rapid and safety test in the same system (da Hora et al., 2019). Recently, people can develop neuronal organoid model mimicking GBM features from induced pluripotent stem cells (iPSC) by mutated c-met gene (Hwang et al., 2020). However, due to the multiple procedures of organoid cultures, it takes around 21–45 days to obtain a mature drug screening organoid (Hubert et al., 2016; Ogawa et al., 2018; Jacob et al., 2020). For the high-grade GBM patients, it is an emergency to make treatment decision within a short time course. Our GSC culture system could be established within only 24 h to 3 days immediately after surgery, which may save some times as compared to the complex organoid culture system.

Personalized medicine therapy is a promising approach for GBM. However, it requires timely selection of appropriate therapeutic targets or anticancer agents. A desired strategy for patients with GBM is to only kill hGSCs but not hNSCs. Therefore, it is necessary to understand the morphological and molecular characteristics of hGSCs. Sox2 is a stem cell marker of adult neurogenesis (Steiner et al., 2006), and nestin is a marker used to examine neurogenesis within the adult brain (Doyle et al., 2001). The high levels of Sox2 and nestin expression observed in hGSCs are indicative of their stemness. GFAP and S100-beta are mature astrocyte markers (Garcia et al., 2004). GFAP expression was low in hGSCs, but surprisingly, the S100-beta expression was high in GSCs. S100 beta was first identified as an astrocyte marker (Castets et al., 1997). Recently, some malignant diseases showed highly expressed S100-beta as well. The importance of understanding the differences between hGSCs and hNSCs was previously noted in clinical treatments as well (Stoyanov et al., 2018). In the present study, we focused to compare several key genes. As expected, we observed higher expression of cell cycle genes and lower expression of p53 tumor suppressor gene expression in hGSCs than that in hNSCs. We have analyzed the 10 GBM-related genes reported recently (Jiang et al., 2020; Larsson et al., 2020; Yang et al., 2020) and six of them showed expression differences between hGSCs and hNSCs, further validating our hGSC derivation approach.

In summary, we have established a fast and efficient protocol to obtain high-purity GBM stem cell culture for both basic research and translational research. The approach detailed here has the very potential to facilitate drug screening directly with patient-relevant cells and enable personalized medicine practice. We hope that similar approaches like ours may 1 day prove to be beneficial to GBM patients.

## Data Availability Statement

The datasets presented in this study can be found in online repositories. The names of the repository/repositories and accession no: prjca004144.

## Ethics Statement

The studies involving human participants were reviewed and approved by Ethics Committee of Shanghai 10th people’s Hospital. The patients/participants provided their written informed consent to participate in this study.

## Author Contributions

ZG, X-XH, and HH conceived and designed the research. X-XH and CC performed sample collection, experiments, and data analysis. L-MY, MW, D-YH, and JR helped perform the cell experiments. M-HZ, L-YZ, W-HZ, and WH performed data analysis. X-XH and CC wrote the manuscript. All authors contributed to the article and approved the submitted version.

### Conflict of Interest

The authors declare that the research was conducted in the absence of any commercial or financial relationships that could be construed as a potential conflict of interest.
